# Digit-only sauropod pes trackways from China – evidence of swimming or a preservational phenomenon?

**DOI:** 10.1038/srep21138

**Published:** 2016-02-18

**Authors:** Lida Xing, Daqing Li, Peter L. Falkingham, Martin G. Lockley, Michael J. Benton, Hendrik Klein, Jianping Zhang, Hao Ran, W. Scott Persons, Hui Dai

**Affiliations:** 1 School of the Earth Sciences and Resources, China University of Geosciences, Beijing, China; 2 Geological Museum of Gansu, Lanzhou 730040, China; 3 Structure and Motion Laboratory, Department of Comparative Biomedical Sciences, Royal Veterinary College, Hatfield AL97TA, UK; 4 Department of Ecology and Evolutionary Biology, Brown University, Providence, RI 02912, USA; 5 Dinosaur Trackers Research Group, University of Colorado, Denver, Colorado, USA; 6 School of Earth Sciences, University of Bristol, Bristol, BS8 1RJ, UK; 7 Saurierwelt Paläontologisches Museum, Alte Richt 7, D-92318 Neumarkt, Germany; 8 Key Laboratory of Ecology of Rare and Endangered Species and Environmental Protection, Ministry of Education, Guilin 541004, China; 9 Department of Biological Sciences, University of Alberta 11455 Saskatchewan Drive, Edmonton, Alberta T6G 2E9, Canada; 10 No. 208 Hydrogeological and Engineering Geological Team, Chongqing Bureau of Geological and Mineral Resource Exploration and Development, Chongqing 400700, China

## Abstract

For more than 70 years unusual sauropod trackways have played a pivotal role in debates about the swimming ability of sauropods. Most claims that sauropods could swim have been based on manus-only or manus-dominated trackways. However none of these incomplete trackways has been entirely convincing, and most have proved to be taphonomic artifacts, either undertracks or the result of differential depth of penetration of manus and pes tracks, but otherwise showed the typical pattern of normal walking trackways. Here we report an assemblage of unusual sauropod tracks from the Lower Cretaceous Hekou Group of Gansu Province, northern China, characterized by the preservation of only the pes claw traces, that we interpret as having been left by walking, not buoyant or swimming, individuals. They are interpreted as the result of animals moving on a soft mud-silt substrate, projecting their claws deeply to register their traces on an underlying sand layer where they gained more grip during progression. Other sauropod walking trackways on the same surface with both pes and manus traces preserved, were probably left earlier on relatively firm substrates that predated the deposition of soft mud and silt . Presently, there is no convincing evidence of swimming sauropods from their trackways, which is not to say that sauropods did not swim at all.

Could sauropods swim? This is a question that has been raised in the literature on multiple occasions since the 1940s, generally based on the occurrence of manus-only or manus-dominated trackways. However, the notion of swimming sauropods has even older roots originating with the archaic, and now largely discredited idea that sauropods were aquatic either to support their massive weight or to escape predators. So were there any sauropods possibly performing an aquatic or semi-aquatic lifestyle, and is there evidence that they were able to swim ? The track literature still continues to raise this question periodically. In 1944, Roland T. Bird suggested the swimming sauropod hypothesis based on a trackway composed almost exclusively of manus prints from the Cretaceous of Texas. Bird suggested that the trackway could have been produced by a sauropod ‘punting’ off the bottom with its forelimbs, while the hindlimbs floated clear of the substrate^1^. In addition, Ishigaki also interpreted manus-only trackways from the Jurassic of Morocco as evidence of partially buoyant or swimming sauropods^2^. However, Lockley and Rice^3^ and Lockley *et al*.^4^ suggested that these manus-only or manus-dominated trackways are simply undertracks, and in the case of the specimens from Texas proved this by showing that Bird’s trackway of a purported swimmer in fact included pes undertracks^5^,^6^. Even so, many manus-only trackways lack corresponding pes undertracks. These aforementioned studies as well as more recent contributions by Falkingham *et al*.^7^,^8^, Lockley *et al*.^9^ and Ishigaki & Matsumoto^10^ demonstrate that manus- or pes-dominated trackways could be formed on a surface, as a result of differential pressures beneath fore- and hind-feet. Put another way, a layer of sediment can, in theory, serve a similar purpose to a column of water in protecting another surface from the full or direct impact of a trackmaker’s foot^3^. Therefore, definitive fossil evidence supporting the ability of sauropods to swim has not yet been found. Nevertheless, other unusual trackway configurations, purportedly indicating manus-only sauropod trackways, have recently been interpreted as evidence of swimming^11^,^12^, and again these interpretations have been rejected 13,14. However, negative evidence does not necessarily imply the general absence of swimming ability or behaviour. Indeed, extant large terrestrial animals such as elephants have been known to swim in both fresh and marine water for considerable distances^15^.

In 2000, workers from the Research Center of Paleontology of the Bureau of Geology and Resource Exploration of Gansu Province discovered ten dinosaur tracksites in a small area representing the Hekou Group in Yanguoxia, Gansu Province^16^,^17^. Six sites were documented in the area. Tracks from sites 1 and 2 are the best preserved and most diverse. In 2002, this area was incorporated into Liujiaxia Dinosaur National Geopark (Fig. 1). Zhang *et al*.^18^ and Li *et al*.^19^ reported a preliminary exploration of these tracksites, based on a large exposure at site 1 and a limited exposure at site 2. Altogether the sites reveal about 2000 exposed tracks, including well-preserved assemblages of dinosaur (theropod, sauropod and ornithopod), pterosaur, and bird tracks^20^,^21^. Most exposures represent the same horizon associated with a fining-upward sandstone sequence culminating in ripple marked siltstones (Fig. 2) and overlain regionally by a thick sequence of floodplain siltstone and mudstone.

Unusual sauropod tracks were found at No. 2 site of Liujiaxia Dinosaur National Geopark (YSII), which preserve only clear claw traces without the impressions of the whole pes or any trace of the associated manus. These occur in a more or less regular trackway pattern, but predominantly show only large pes claw traces (Fig. 3). In a preliminary study, Zhang *et al*.^18^ suggested that the trackmaker was walking on a higher or overlying stratigraphic layer, through which only the pes claws penetrated. Li *et al*.^19^ proposed an alternate theory that the “trackmakers were sauropods that walked in water on tip toes of their hind feet”. Note here the aforementioned implication that either water or a layer of sediment may support or “buoy up” a trackmaker to the extent that it does not fully register its footprints on a given surface^3^: i.e., two different interpretations can potentially explain the same feature. However, these sauropod tracks have yet to be described and discussed in detail. During 2014 to 2015, the senior authors were invited by the Liujiaxia Dinosaurs National Geopark and the Geological Museum of Gansu to study dinosaur tracks in Liujiaxia Dinosaur National Geopark. During that period, we found more claw-only sauropod tracks which are described and discussed in detail below.

## Geological setting

Liujiaxia Dinosaur National Geopark lies 54 km west of the city of Lanzhou, on the north shore of Taiji Lake (Yanguoxia Reservoir), Yongjing County, Linxia Hui Autonomous Prefecture, Gansu Province. The track locality is positioned at the southeastern edge of the Lanzhou−Minhe Basin.

The Lower Cretaceous Hekou Group at Lanzhou-Minhe Basin is a continental clastic deposit of great thickness^22^. The Early Cretaceous age is based on biostratigraphical evidence such as bivalves, ostracods, fishes^23^, dinosaur skeletal fossils^24^, and tracks^18^,^25^. Ji *et al*.^26^ suggested that the upper part of the Hekou Group was deposited approximately in the Aptian–Albian stages based on characteristics of pollen assemblages. Tang *et al*.^27^ dated the middle and lower parts of the Hekou Group to 140.66–124.19 Ma (Berriasian – Aptian) according to paleomagnetic data.

In 1997, the Hekou Group was divided into eight informal formation-level units^28^,^29^,^30^. The tracks of the Yanguoxia area, at Liujiaxia Dinosaur National Geopark, are from the 6th informal formation-level unit of the Hekou Group^25^,^31^. This unit is a semi-deep lake facies deposit and comprises purplish red thickly-bedded massive silty mudstones interbedded with thickly-bedded fine sandstones and occasionally grayish green thin-bedded silty mudstones with ripple bedding and invertebrate traces in the sandstone^31^. This unit contains numerous bands of grayish green and grayish yellow medium- to thick-bedded mudstone, marlstone and micrite with single bed thickness of about 0.3–0.4 m, indicating maximum flooding surfaces in the middle and upper parts of the Hekou Group^31^.

Deposits producing tracks in the Yanguoxia Region consist of purple mudstone and silty mudstone interbedded with fine sandstone. Blue grey silty mudstone beds are commonly seen in upper parts of these deposits. A number of ripple beds, parallel beds and asymmetric ripple marks are seen in the sandstone with rain prints, mud cracks and invertebrate traces on the surface, suggesting a depositional environment typical of shallow shore lacustrine facies^31^. Tracks are between bands of grayish green siltstone-mudstone and on the surface of the purplish red fine sandstone. The number 1 and number 2 Liujiaxia Dinosaur National Geopark tracksites occur at the top of a relatively thick sandstone sequence, fining up at the top and overlain by the aforementioned fine purplish-red siltstones and mudstones. The track-bearing layers are exposed on a dip slope (dip about 20°–25°) as the result of the local removal of most of the overlying siltstones and mudstones by down-dip erosion into the adjacent valley. These overlying siltstones and mudstones are well exposed in the valley^32^.

## Materials and Methods

**Figure 1:**
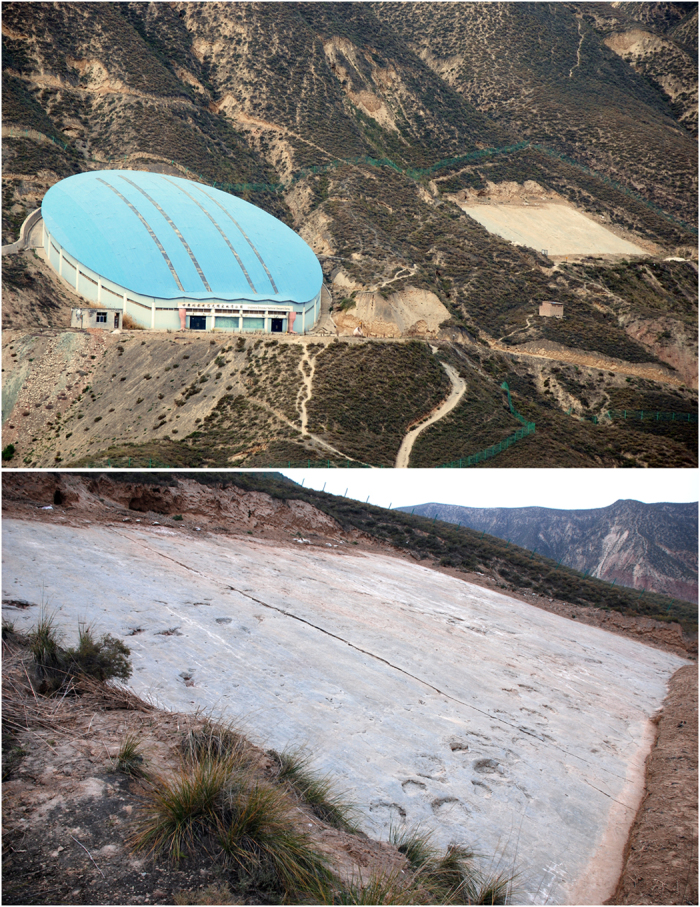
Photographs with Yanguoxia No. 1 and 2 tracksites (top), and outcrop of No. 2 tracksite (bottom) (Photographs by L.X.).

**Figure 3:**
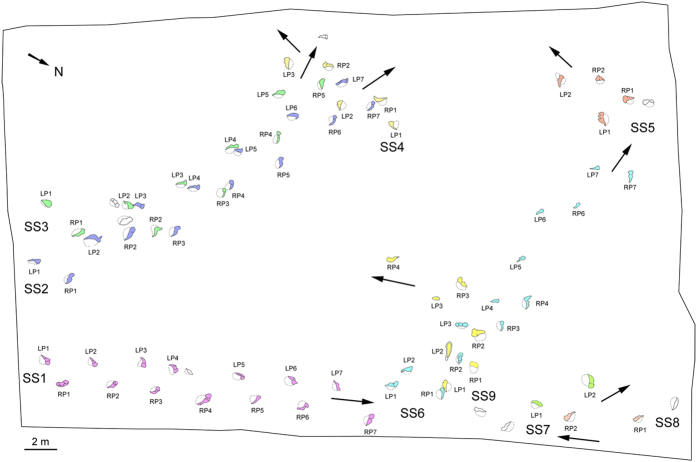
Map with distribution of digit-only sauropod trackways at Yanguoxia No. 2 tracksite. (Line drawing by L.X.).

The No. 2 tracksite was excavated to expose an area of about 1060 m^2^ (Figs 1 and 3) which is much larger than the area exposed prior to the work reported by Zhang *et al*.^18^. Every footprint was numbered and outlined with chalk. Then, the site was covered by large transparent plastic sheets, on which the outlines of the tracks were traced.

For calculation of hip heights and speed estimates, the methods of Alexander^33^ and Thulborn^34^ were adopted. The different values estimated from these different methods were compared but are not discussed further.

Photogrammetric models were produced from multiple digital photographs (Canon EOS 5D Mark III), which were converted into scaled 3D textured mesh models using Agisoft Photoscan^35^,^36^. The mesh models were then imported into Cloud Compare, where the models were rendered with scaled color topographic profiles.

## Footprint Morphology

The Yanguoxia No. 2 tracksite preserves at least nine large-sized digit-only pes trackways, catalogued as YSII-SS1−SS9 (SS = special sauropod trackway) (Supplementary material 1). Trackways YSII-SS1−SS9 contains 14, 14, 10, 5, 4, 13, 2, 2 and 7 tracks respectively. Furthermore eight additional isolated tracks are present. The lengths of most pes tracks are between 16.5–48 cm with a mean length of 27.4 cm and a mean L/W ratio of 0.4. All of the original tracks remain in the field at YSII. It should also be noted that this same tracksite surface reveals sauropod trackways with a typical mode of preservation, showing both manus and pes with clear outlines of the complete tracks.

**Figure 4:**
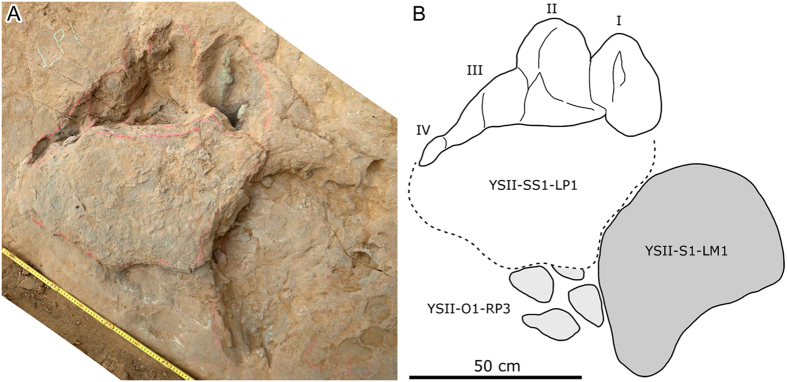
Photograph (**A**) and outline drawing (**B**) of the best-preserved digit-only sauropod track YSII-SS1-LP1, associated with the manus track of another sauropod YSII-S1-LM1 and an ornithopod pes track YSII-O1-RP3. (Photographs and line drawing by L.X.).

Among all the pes-only trackways, the best preserved is YSII-SS1(Supplementary material 2). In YSII-SS1-LP1 (LP = left pes tracks) (Fig. 4), for example, digit traces I, II and III of most of the well-preserved pes traces (except RP1, RP = right pes tracks]) have recognizable claw marks, digit IV has small nail marks or depressions made by small unguals or foot callosities. The digit I impression is the most developed (deepest) and oval in shape (length greater than width). The impression of digit II is also oval in shape (length greater than width), slightly longer than or subequal to the digit I impression and runs deep into the sediment proximo-laterally. The digit III impression also increases in depth laterally. It is approximately 1/2 to 1/3 the length of the impressions left by digit I and II. Digit IV produced an exceptionally small oval impression, only seen in some tracks. The average outward rotation of the pes from the midline of YSII-SS1 is 40°.

Lingulate sand mounds are preserved at the posterior end of the all the pes traces in YSII-SS1 (except RP1), showing that the substrate was pushed up posteriorly behind the deeply impressed digits. This configuration is a common feature of swim tracks^37^, including those of theropods^20^,^38^ and turtle tracks^9^,^32^, but mostly occurs in irregular configuration not in regular walking sequences.

The other trackways are similar to YSII-SS1 in their morphology. Digit I impression of SS2-LP2 has sand mounds projecting distally while SS5-LP1 shows evident dragging marks extending anteriorly.

## Comparisons with other sauropod trackways

**Figure 5:**
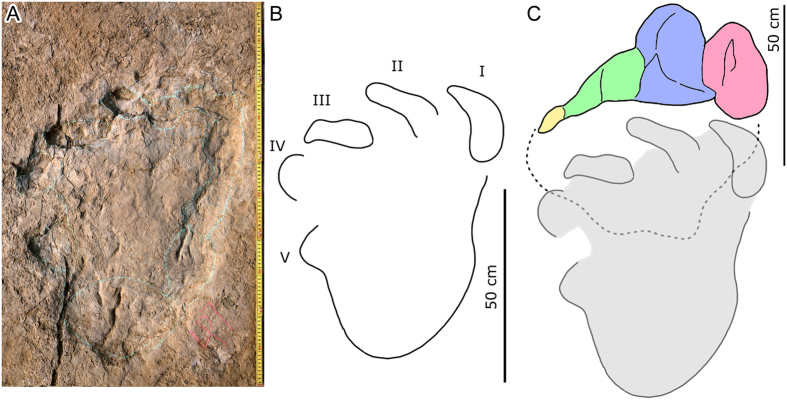
Photograph (**A**) and outline drawing (**B**) of the best-preserved sauropod track YSII-S3-LP7, and comparison with YSII-S3-LP7 and YSII-SS1-LP1 (**C**). (Photographs and line drawing by L.X.).

Most sauropod trackways in China are wide- (or medium-) gauge and are therefore referred to the ichnogenus *Brontopodus*^39^,^40^,^41^,^42^,^43^. In addition to the YSII-SS1–SS9 trackways, the Yanguoxia No. 2 tracksite displays at least a further five sauropod trackways that show the typical pattern with both manus and pes imprints preserved. Furthermore there are four sauropod trackways, also with typical manus - pes preservation, at the Yanguoxia No. 1 tracksite (YSI), 120 m southeast of No. 2 tracksite. Other than the pes-only trackways from site 2, most sauropod trackways from the Yanguoxia No. 1 and 2 tracksites have basically the same imprint morphology and trackway pattern. The most complete tracks YSII-S3-LP7 and LM7 (LM = left manus tracks) (Fig. 5) and the corresponding trackway, appear to show all the features of a typical sauropod trackway such as the *Brontopodus*-type: wide−gauge, pes tracks longer than broad, with large, outwardly directed claw marks of digits I–III, a small digit IV trace and small callosity or pad mark representing digit V. Manus tracks are semicircular to U−shaped with rounded posteromedial/posterolateral traces of digits I and V, respectively^44^,^45^. In YSII-S3-LP7 and YSII-SS1-LP1, the arrangement of digit traces I–IV is quite similar, demonstrating unequivocally that YSII-SS1–SS9 trackways were made by a sauropod trackmaker. Of note, all the digit marks of the YSII-SS1–SS9 tracks entered the sediment vertically, to create deep inverted cone-shaped impressions. These kinds of tracks are obviously different from those in typical sauropod trackways.

## Trackmaker

The wide gauge pattern of the *Brontopodus*-type trackways (*sensu* Marty^40^) suggests that the tracks were left by titanosauriform sauropods^39^,^46^. Currently, three large titanosauriform sauropods have been identified from the Hekou Group of the Lanzhou−Minhe Basin: *Huanghetitan liujiaxiaensis*^47^, *Daxiatitan binglingi*^48^, and *Yongjinglong datangi*^49^. These three Hekou Group sauropods were members of the same fauna, but each is distinct and has numerous autapomorphies^49^. *Daxiatitan* has femora with features similar to *Opisthocoelicaudia*^50^: outwardly angled femora, eccentric femoral mid-shaft cross-section, and dorso-medially beveled femoral distal condyles; You *et al*.^48^ suggest some affinity of *Daxiatitan* to sauropod tracks from the Yanguoxia site due to these characteristics. Thus, a good case can be made that trackmakers at the Yanguoxia sitemight possibly be referred to titanosauriform sauropods whose skeletal remains occur in the same strata and indicate wide-gauge forms. However, the absence of forefeet and hindfeet in these sauropod skeletal fossils prevents further comparison with the tracks. On the other hand, the absence of manus traces in the YSII-SS trackways can be hardly explained when considering the anterior shift of weight and center of mass (CoM) present in titanosauriform sauropods. So, a final attribution and interpretation of the trackmaker cannot be given here.

## Locomotion and footprint registration

The Yanguoxia No. 2 tracksite yields rich theropod, sauropod and ornithopod trackways^18^. Overlap of these tracks may reflect the sequence in which trackmakers traversed this surface. YSII-SS1-LP1, for example, covers part of an ornithopod pes track (YSII-O1-RP3) with a sand mound which, on the posterior side, is in contact with the edge of another sauropod manus impression (YSII-S1-LM1) (Fig. 4). The sequence therefore indicates that the ornithopod track (YSII-O1), was made before either of the sauropod tracks (YSII-S1 and YSII-SS1).

Although Li *et al*.^19^ suggested that YSII-SS1 shows some very faint associated manus impressions, subsequent investigations fail to confirm this. Whilst the same surface indeed shows some extremely shallow oval imprints, they are not in positions consistent with theYSII-SS1 pes tracks and are therefore unlikely to be associated with this trackway. These imprints may be undertracks (*sensu* Marty 40) of sauropod manus impressions produced at a higher level^3^. Alternatively, they may have been produced at a later time, when the substrate was more firm and dry, by animals that exerted a higher underfoot pressure beneath the manus compared with the pes^7^,^51^.

Based on the incompletely exposed Yanguoxia No. 2 tracksite, Zhang *et al*.^18^ suggested that the YSII-SS1 trackway was formed by an “accelerating sauropod” although the pattern does not show a “regular lengthening of stride”. However, the changes in stride length are subtle and not marked or regular enough to indicate a pronounced increase in speed.

Li *et al*.^19^ suggested that YSII-SS1was left by a trackmaker getting out of the water to approach the lake-shore. However, in this scenario we would expect a continuous morphological change along the trackway, for instance a deepening or shallowing of the tracks, or increasing presence of manus impressions, as the substrate saturation changed or the water became shallower and buoyancy decreased. However, neither of these features is observable in the trackway, meaning that if the animal was buoyed by water, the water depth would need to have been consistent over the length of the exposed tracks exhibiting this morphology.

**Figure 6:**
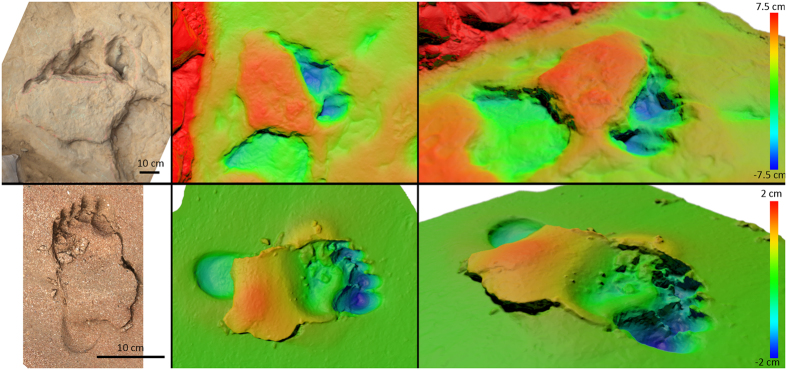
Sauropod (top) and human (bottom) tracks with push-back structures behind the digits, presented as phototextured and height-mapped models. The human footprint is of an adult female walking on moist beach sand. The digits have sunk deeply during kick-off, causing sediment immediately behind them to be pushed upwards and backwards. While a heel impression is visible, it is relatively shallow in comparison to the fore-foot. We propose a similar mechanism was involved in the formation of digit-only sauropod tracks such as YII-SS1-LP1 (pictured here). (Photographs and photogrammetric models by P.F.)

Henderson^52^ used 3D computer modeling to demonstrate that some floating sauropods such as *Diplodocus* and *Apatosaurus*, which possessed posterior centers of mass (CoM) could have produced pes-only tracks. But *Brachiosaurus* belonging to Titanosauriformes and hypothesized to have had a more anterior CoM, would have ‘tipped’ forwards, meaning that when partially buoyant it would have left manus-only tracks: see Lockley^53^,^54^,^55^ for further observations on sauropod anterior-posterior CoM and implications for trackway characteristics. Given that the tracks discussed here are attributed to titanosauriform dinosaurs based on pedal morphology and wide trackway gauge, we would expect the trackmakers to have an anteriorly positioned CoM, which would have favored manus-dominated trackway formation if partially buoyed in water, but also in fully terrestrial locomotion, if the manus was impressed deeper than the pes and preserved on a lower layer. However, the Yanguoxia trackways discussed here are pes-dominated, and predominantly record only deep claw marks of the pes. This morphology is consistent with strong penetration of the unguals during the “kick-off” phase of trackmaking^34^. To demonstrate this, we illustrate a human footprint formed by a female running on firm, moist, beach sand (Fig. 6). In this case, the foot as a whole failed to impress the substrate deeply, but when the kick-off pressure increased and exceeded the bearing capacity of the substrate, the toes sank more deeply^51^ and a lingulate-shaped sand wedge was displaced posteriorly. At this point, the ground reaction force is directed forwards^56^, which causes the sand to easily shear posteriorly, resulting in the lingulate displacement of sediment behind the digits. This human trackway contained multiple tracks exhibiting this morphology. The similarities between the human footprint and the sauropod tracks discussed here strongly suggest that these tracks are not the result of a buoyant sauropod dabbling its toes on a lake bottom, but rather one registering tracks on a firm sub-aerial sandy substrate, although as discussed below, not necessarily one without some additional superficial sediment layers. That multiple trackways record this formational process (YSII-SS1 to YSII-SS9) indicates that either the sediment surface remained relatively consistent in moisture content for some time, or that the trackways were formed more-or-less contemporaneously. Because this phenomenon is not observed for the manus (which left no visible impression), we may infer that only underfoot pressures beneath the pes reached a sufficient peak to overcome the bearing capacity of the substrate.

Here it should be noted that the displaced wedge of sand is a reflection of foot registration dynamics and substrate conditions, especially posteriorly directed force during kick off. Such displaced sand wedges, referred to as “sand crescents” occur behind mammaloid tracks in Permian through Mesozoic sand dune facies^57^,^58^. They may indicate a slight moisture content and cohesion to the substrate. Similar sand wedges occur behind swim tracks, as in the case of *Kyuangyuanpus* a possible crocodilian track^59^. Thus, the displacement is not characteristic of any particular trackmaker type or paleoenvironment but can occur in many different track-making situations where backward force is applied during foot registration.

## Reinterpreting the pes-only sauropod trackways

Zhang *et al*.^18^ stressed that “ the track maker was walking on a higher or overlying stratigraphic layer, through which only the pes claws penetrated” and further inferred (op. cit. p. 53–54) that “the irregular pes-only sauropod trackway is … evidence of an animal walking on an overlying surface, now removed. The track maker dug its longer claws (pes digits I–III) into the soft underlayers. Assuming the overlying layer to have been soft, as indicated by the depth of the claw impressions in the layer beneath, the irregular trackway may have been the result of the large animal varying its gait to negotiate a difficult and potentially dangerous surface where it may have run the risk of slipping or getting stuck. The irregular trackway, with traces of spread or projecting claws could be interpreted as evidence of such a reaction to an unstable substrate” These inferences require further evaluation in the light of the newly exposed evidence available for the present study.

At the time when Zhang *et al*.^18^ first investigated the Yangouxia site No 2, only a small area was exposed (op. cit., Fig. 4) including the portion of the surface with trackway YSII-SS1. Now eight trackways in the SS category are exposed. This is evidence that conditions suitable for the registration and preservation of pes-only sauropod trackways were consistent over the much larger area now available for study. For pes-only and manus-only sauropod trackways, respectively, in Cretaceous rocks, Falkingham *et al*.^60^ observed that “manus-dominated tracks are most commonly recorded in cohesive substrates (e.g. mudstones), whereas pes-dominated tracks tend to be restricted to non-cohesive substrates (e.g. sandstones).” It is also important to note that, although both pes-only and typical manus-pes sauropod trackways are registered on the same surface, this does not necessarily imply that they were made at the same time, or as noted below, that the whole track was even registered on the same surface. In short, we need to consider plausible interpretations to explain two such different modes of preservation of sauropod tracks on the same surface. Here it is important to note that the typical site 2 sauropod trackways (with manus and pes traces) are more or less consistent in depth and flat-bottomed, without the unusually deep sub-vertically oriented claw traces in the pes-only tracks. This suggests that the substrate was fairly firm, i.e., not saturated at the time of the registration of the typical tracks. It also suggests that the typical tracks are true tracks, not undertracks. It is reasonable to infer that the tracks on the present sandstone surface would not be well preserved if any significant thickness of sediment were overlying it at the time of track registration. By contrast, as noted by Zhang *et al*. 17, and consistent with this study, the pes-only sauropod tracks are deep, with steeply penetrating claw traces. This implies both a moister, more saturated substrate and an overlying layer of sediment on which the animals were walking. These conditions are easily and quite simply explained by the fact that the track-bearing sandstone layers are overlain by fine mudstones and siltstones indicating a transition to subaqueous (lacustrine) deposition. As noted above, these overlying siltstones and mudstones are well exposed in the area^32^. Sauropods walking on a layer of soft silt and mud deposited on top of the sandstone would likely spread their pes claws to grip and penetrate the substrate steeply: i.e., the lack of a firm substrate would not require the lateral folding of the pes claws typically seen in shallower tracks made on firm substrates that would have been more difficult to penetrate deeply. The lack of preserved manus traces is also consistent with this interpretation, because the sediment above the sand layer would prevent their registration. Moreover, as sauropod manus claws (unguals) especially II-V are short they could not penetrate superficial layers deeply as done by the large pes claws.

It is important to note that in this interpretation the pes-only sauropod tracks are not undertracks transmitted through layers of overlying sediment, but they are actually true tracks, albeit only representing the ungual (claw) portions of the foot, that penetrated the overlying mud-silt sediment and directly registered in the sand: see Thulborn^34^ for comments on how these differ from undertracks. Due to the successful penetration of the pes claws into the sand, and the failure of the manus tracks to impact this level, the pes would have gained greater purchase allowing for a robust posterior “kick off” motion as the animal progressed forward. Such motion could easily have created the lingulate sand displacements seen in these tracks. In fact, unless the sand were covered by a clay drape, it is difficult to conceive how the lingulate sand displacement features could be preserved if they were exposed sub-aerially on a surface that was then inundated by mud and silt depositing waters.

Given the speed estimates derived from the trackways and the fact that sauropods were graviportal, they, unlike our human subject, probably could not run. On the other hand, recent elephants of similar proportions are able to run, and the same may have been true also for very small juvenile sauropods for example. The latter, however, cannot be proved by trackways presently. In the scenario where their main purchase was on a buried substrate layer with their toe tips, the posterior force exerted would be similar to that made by an individual that was running and exerting force in the kick-off phase.

We note that despite proposing the above arguments other scenerios might be possible. The difficulties with proposing an unambiguous interpretation are that the sedimentological history of the deposition of the sandstone and overlying silts and muds cannot be known with certainty. Deposition rates, levels of sediment saturation and duration of substrate exposure to subaerial or subaqueous conditions cannot be determined precisely even with detailed sedimentological analysis.

As noted above, there is, as yet, no convincing trackway evidence for swimming sauropods, despite many attempts to promote this scenario based on manus-only or manus-dominated trackways. Given the arguments above, the rare occurrence of pes-only trackways also fails to provide a convincing argument for a swimming scenario. As noted below, the speed estimates derived from these trackways also gives no support for a swimming sauropod scenario.

## Speed

The typical sauropod tracks on the same surface as the pes-only sauropod tracks have a typical L/W (length/width) ratio of 1.3 (as YSII-S3). The width of pes-only sauropod tracks YSII-SS1 (average 64.7 cm) is nearly equal to that of the walking sauropod tracks from the same surface. Track length of YSII-SS1 (84.1 cm) can be roughly figured out by using the L/W ratio of the latter. A foot length/hip height ratio for a sauropod is 4.0^33^ or 5.9^34^. The relative stride length ratios of the YSII-SS1 trackway is between 0.6–0.88 and accordingly suggest walking. Using the equation to estimate speed from trackways^33^, the mean locomotion speed of the trackmaker is between 2.66–4.21 km/h. These data are basically the same as those of typical sauropod trackways^61^. This suggests a swimming scenario is not plausible. In fact, most previous swim-track hypotheses have been rejected on the simple grounds that the trackway pattern is typical of sauropod walking locomotion.

## Digit-only ornithopod and small-sized sauropod tracks

The digit-only ornithopod trackways that are also found at the No. 1 and No. 2 tracksites, as well as small-sized sauropod trackways, indicate similar preservation pattern. The digit-only ornithopod trackways include at least five trackways at No. 1 tracksite of Liujiaxia Dinosaur National Geopark and 49 trackways from No. 2 tracksite. There are only two small digit-only sauropod trackways from the No. 2 tracksite.

**Figure 7:**
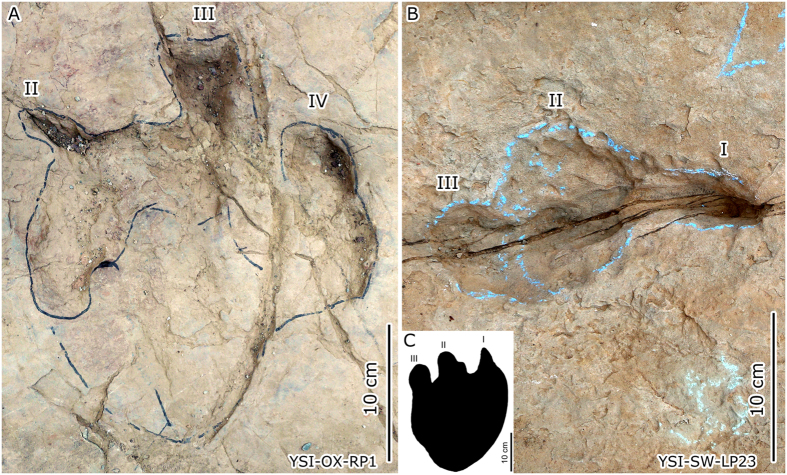
Photograph of digit-only ornithopod track YSI-OX-RP1 (**A**) and small-sized sauropod track YSI-SW-LP23 (**B**), and the comparison with small-sized sauropod track from Litan site (**C**). (Photographs and line drawing by L.X.).

Fujita *et al*.^62^ considered these digit-only ornithopod trackways from Yanguoxia No. 2 tracksite as those made while the animals were partially submerged, propelling themselves by toe-only steps, leaving subaqueously registered trails as proposed by Gierliński^63^ but challenged by Lockley^5^. The purported evidence includes backward drag marks at the rear of the tracks, or sand mounds preserved at the posterior margins of some pes traces. After examining digit-only ornithopod tracks carefully, we observe examples with shallow heel traces at the No. 1 tracksite. YSI-OX (No. X ornithopod trackway) has about 23 tracks with extremely shallow heels in 8 tracks and much deeper digit impressions (Fig. 7). Such morphology is similar to the human footprints mentioned above.

Moreover, Fujita *et al*.^56^ also mentioned tail traces associated with digit-only ornithopod trackways, but this is not a common finding in all 54 trackways. If the trackmakers had consistent stances, such tail traces would be common. Therefore, further analysis is required to ensure that these traces were not water-created features.

Compared with the 54 narrow-gauge ornithopod trackways, the two wide-gauge small-sized sauropod trackways at No. 2 tracksite only preserve digit impressions (YSI-SW [No. w sauropod trackway] and YSI-SV [No. v sauropod trackway]). They are morphologically almost the same as the anterior parts of the small-sized sauropod pes track from the Litan site, Yanguoxia^24^, indicating that YSI-SW and YSI-SV have a strong affinity with the Litan track.

## Conclusions

We report an assemblage of nine sauropod trackways consisting of digit-only pes tracks from the Lower Cretaceous Hekou Group of Gansu.

To date this is the only site preserving this type of trackway.

The trackway pattern is similar to that found in trackways of typical walking sauropods except that the toe impressions are much deeper.

The tracks also show a displaced wedge of sand similar to that commonly found in other tracks made in sandy substrates.

The only other well-known category of unusual incomplete sauropod trackway, that of manus-only or manus-dominated trackways, has been the subject of much debate. Previously many authors have interpreted these as evidence of swimming. However, others have disputed these claims and interpreted manus-only trackways as undertracks or examples of unusual preservation that cannot be used to support the swimming sauropod scenario.

The digit-only pes sauropod trackways are here interpreted as evidence of animals walking on a silt and mud substrate above the sandstone layer into which the pes claws penetrated from above.

Thus the digit-only pes trackways have a preservational explanation and are not evidence of swimming or unusual behavior.

## Additional Information

**How to cite this article**: Xing, L. *et al*. Digit-only sauropod pes trackways from China - evidence of swimming or a preservational phenomenon?. *Sci. Rep.* **6**, 21138; doi: 10.1038/srep21138 (2016).

## Supplementary Material

Supplementary Information

Supplementary Information

## Figures and Tables

**Figure 2:**
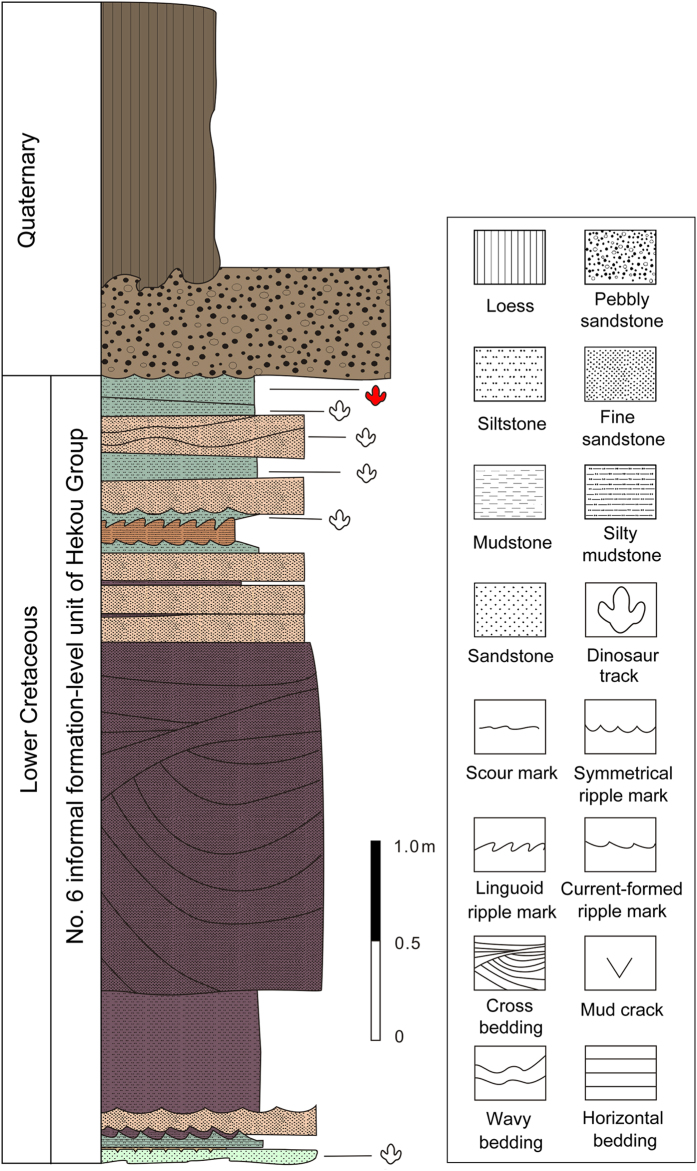
Stratigraphic section of sedimentary sequences in the Yanguoxia area with position of the footprint level. (Line drawing by L.X.).
