# Supplementary figures and images for: Digit-only sauropod pes trackways from China – evidence of swimming or a preservational phenomenon?
Source: Sci Rep. 2016 Feb 18;6:21138. doi: 10.1038/srep21138 (PMC4758031; doi:10.1038/srep21138)

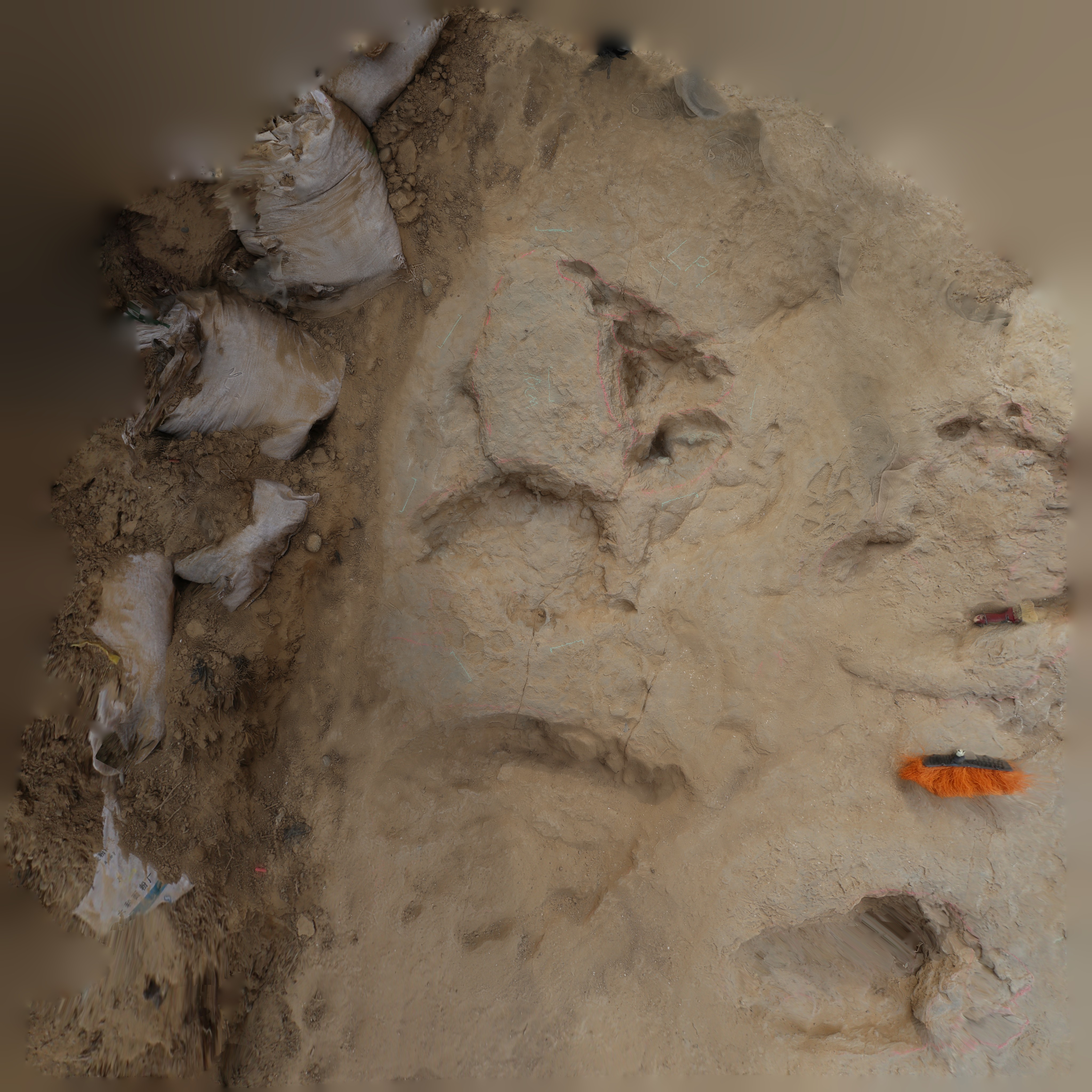

Supplement: Supplementary Information [file srep21138-s1.zip › model/20150927Lida_1.jpg]
