# Supplementary material for: Digit-only sauropod pes trackways from China – evidence of swimming or a preservational phenomenon?
Source: Sci Rep. 2016 Feb 18;6:21138. doi: 10.1038/srep21138 (PMC4758031; doi:10.1038/srep21138)

Digit-only sauropod pes trackways from China – evidence of swimming or a preservational phenomenon?

Lida Xing[1], Daqing Li[2], Peter L. Falkingham[3, 4], Martin G. Lockley[5], Michael J. Benton[6], Hendrik Klein[7], Jianping Zhang[1], Hao Ran[8], W. Scott Persons IV[9], Hui Dai[10]

[1]School of the Earth Sciences and Resources, China University of Geosciences, Beijing, China; [2]Geological Museum of Gansu, Lanzhou 730040, China; [3]Structure and Motion Laboratory, Department of Comparative Biomedical Sciences, Royal Veterinary College, Hatfield AL97TA, UK; [4]Department of Ecology and Evolutionary Biology, Brown University, Providence, RI 02912, USA; [5]Dinosaur Trackers Research Group, University of Colorado, Denver, Colorado, USA; [6]School of Earth Sciences, University of Bristol, Bristol, BS8 1RJ, UK; [7]Saurierwelt Paläontologisches Museum, Alte Richt 7, D-92318 Neumarkt, Germany; [8]Key Laboratory of Ecology of Rare and Endangered Species and Environmental Protection, Ministry of Education, Guilin 541004, China; [9]Department of Biological Sciences, University of Alberta 11455 Saskatchewan Drive, Edmonton, Alberta T6G 2E9, Canada; [10]No. 208 Hydrogeological and Engineering Geological Team, Chongqing Bureau of Geological and Mineral Resource Exploration and Development, Chongqing 400700, China.

Supplementary material. 1. Measurements (in cm) of sauropod tracks from Yanguoxia No. 2 tracksite, Gansu, China.

Number	L	W	D	M’L	M’D	L/W	PL	SL	TW	PA	R
YSII-SS1-LP1	31.0	74.0	13.4	39.0	4.7	0.4	176.0	284.0	221.3	101	29
YSII-SS1-RP1	22.0	34.0	3.7	—	—	0.6	192.0	295.0	—	104	69
YSII-SS1-LP2	25.0	66.0	13.8	35.0	0.7	0.4	181.0	285.0	212.6	101	27
YSII-SS1-RP2	22.0	67.0	11.3	25.0	1.3	0.3	187.0	246.0	—	85	48
YSII-SS1-LP3	24.0	65.0	7.9	30.0	1.1	0.4	176.0	220.0	223.1	82	22
YSII-SS1-RP3	16.5	64.5	8.6	20.0	1.4	0.3	160.0	321.0	—	98	39
YSII-SS1-LP4	26.0	63.0	12.1	29.0	1.5	0.4	256.0	400.0	299.1	106	30
YSII-SS1-RP4	30.0	81.0	14.6	48.0	1.8	0.4	244.0	324.0	—	100	50
YSII-SS1-LP5	28.0	62.0	13.7	42.0	1.3	0.5	176.0	318.0	211.6	101	40
YSII-SS1-RP5	23.0	71.0	13.6	48.0	2.8	0.3	234.0	292.0	—	91	31
YSII-SS1-LP6	27.0	65.0	12.1	49.0	0.9	0.4	172.0	288.0	208.4	93	36
YSII-SS1-RP6	19.0	67.0	12.3	36.5	2.2	0.3	223.0	—	—	—	60
YSII-SS1-LP7	17.5	61.0	11.3	22.0	—	0.3	—	—	336.9	—	—
Mean	23.9	64.7	11.4	35.3	1.8	0.4	198.1	297.5	244.7	97	40
YSII-SS2-LP1	41.0	83.0	17.0	20.0	3.4	0.5	215.0	377.0	265.2	102	52
YSII-SS2-RP1	36.0	75.0	14.2	17.5	2.2	0.5	268.0	433.0	—	119	55
YSII-SS2-LP2	36.0	88.0	11.4	12.0	5.2	0.4	235.0	333.0	249.3	95	31

YSII-SS2-RP2	26.0	59.0	10.5	24.0	1.3	0.4	215.0	313.0	—	80	23
YSII-SS2-LP3	26.0	55.0	11.3	—	—	0.5	270.0	358.0	302.7	88	46
YSII-SS2-RP3	26.0	79.0	11.0	37.0	0.9	0.3	246.0	391.0	—	115	57
YSII-SS2-LP4	26.0	67.0	11.8	26.0	1.2	0.4	216.0	363.0	258.8	112	41
YSII-SS2-RP4	26.0	54.0	13.6	28.0	1.7	0.5	222.0	342.0	—	95	12
YSII-SS2-LP5	28.0	55.0	13.6	37.0	1.2	0.5	240.0	401.0	294.5	100	45
YSII-SS2-RP5	25.0	57.0	11.8	34.0	1.1	0.4	283.0	428.0	—	109	35
YSII-SS2-LP6	35.0	64.0	11.2	32.0	0.7	0.5	243.0	350.0	272.4	98	35
YSII-SS2-RP6	19.0	48.0	8.3	24.0	0.7	0.4	220.0	230.0	—	64	32
YSII-SS2-LP7	24.0	71.0	8.6	35.0	3.5	0.3	215.0	—	249.4	—	—
YSII-SS2-RP7	22.0	68.0	10.7	22.0	1.5	0.3	—	—	—	—	—
Mean	28.3	65.9	11.8	26.8	1.9	0.4	237.5	359.9	270.3	98	39
YSII-SS3-LP1	41.0	87.0	21.0	21.0	5.5	0.5	268.0	501.0	285.8	110	44
YSII-SS3-RP1	32.0	83.0	16.6	21.0	4.2	0.4	343.0	450.0	—	111	60
YSII-SS3-LP2	29.0	64.0	10.1	36.0	3.6	0.5	194.0	330.0	283.1	75	16
YSII-SS3-RP2	31.0	86.0	14.0	27.0	0.6	0.4	323.0	485.0	—	114	78
YSII-SS3-LP3	29.0	74.0	13.8	27.0	1.8	0.4	253.0	402.0	294.2	97	44
YSII-SS3-RP3	24.0	55.0	12.3	39.0	2.1	0.4	282.0	482.0	—	123	53
YSII-SS3-LP4	25.0	69.0	11.7	30.0	1.2	0.4	266.0	425.0	314.6	110	44
YSII-SS3-RP4	25.0	68.0	11.2	25.0	1.1	0.4	252.0	417.0	—	111	45
YSII-SS3-LP5	27.0	67.0	11.3	28.0	1.1	0.4	253.0	—	304.2	—	—
YSII-SS3-RP5	29.0	57.0	11.7	17.0	3.3	0.5	—	—	—	—	—
Mean	29.2	71.0	13.4	27.1	2.5	0.4	270.4	436.5	296.4	106	48
YSII-SS4-LP1	35.0	74.0	9.6	35.0	0.7	0.5	170.0	346.0	203.8	112	52
YSII-SS4-RP1	36.0	66.0	12.6	40.0	2.2	0.5	244.0	385.0		107	27
YSII-SS4-LP2	28.0	67.0	13.6	38.0	3.5	0.4	236.0	408.0	263.8	116	31
YSII-SS4-RP2	26.0	71.0	14.2	31.0	4.0	0.4	245.0	—	—	—	—
YSII-SS4-LP3	28.0	68.0	12.2	30.0	1.0	0.4	—	—	—	—	—
Mean	30.6	69.2	12.4	34.8	2.3	0.4	223.8	379.7	233.8	112	37
YSII-SS5-RP1	38.5	86.0	9.5	29.0	3.3	0.4	183.0	218.0	245.8	62	—
YSII-SS5-LP1	30.0	68.0	12.4	25.0	0.6	0.4	232.0	338.0	—	98	41
YSII-SS5-RP2	24.5	49.0	7.0	28.5	2.8	0.5	214.0	—	277.2	—	—
YSII-SS5-LP2	30.0	75.0	12.3	24.0	2.4	0.4	—	—	—	—	38
Mean	30.8	69.5	10.3	26.6	2.3	0.4	209.7	278.0	261.5	80	40
YSII-SS6-LP1	24.5	62.0	7.5	21.0	2.5	0.4	299.0	198.0	355.4	41	45
YSII-SS6-RP1	19.0	69.0	7.8	31.0	0.2	0.3	198.0	219.0	—	54	67
YSII-SS6-LP2	40.0	84.0	16.0	26.0	3.3	0.5	264.0	378.0	354.5	110	47
YSII-SS6-RP2	21.0	64.0	8.5	12.0	1.0	0.3	195.0	332.0	—	101	31
YSII-SS6-LP3	20.0	47.5	7.5	11.0	0.4	0.4	234.0	246.0	281.4	76	55
YSII-SS6-RP3	18.0	51.0	6.0	15.5	0.5	0.4	153.0	211.0	—	72	42
YSII-SS6-LP4	21.5	36.5	5.6	—	—	0.6	200.0	319.0	221.3	88	31
YSII-SS6-RP4	35.0	76.0	11.5	21.0	0.7	0.5	257.0	—	—	—	—
YSII-SS6-LP5	19.5	62.0	10.7	—	—	0.3		340.0	—	—	43
YSII-SS6-RP5	—	—	—	—	—	—	—	—	—	—	—
YSII-SS6-LP6	21.0	63.0	10.5	16.5	4.2	0.3	218.0	393.0	236.7	119	81
YSII-SS6-RP6	17.5	47.0	5.7	—	—	0.4	237.0	374.0	—	107	70
YSII-SS6-LP7	29.0	57.0	3.8	—	—	0.5	228.0	—	257.5	—	—
YSII-SS6-RP7	24.0	70.0	4.6	—	—	0.3	—	—	—	—	—

Mean	23.8	60.7	8.1	19.3	1.6	0.4	225.7	301.0	284.5	85	51
YSII-SS7-LP1	24.0	67.5	10.3	30.0	3.1	0.4	357.0	—	—	—	44
YSII-SS7-LP2	28.0	67.0	10.3	30.0	2.2	0.4	—	—	—	—	—
Mean	26.0	67.3	10.3	30.0	2.7	0.4	357.0	—	—	—	44
YSII-SS8-RP1	21.0	57.0	7.6	21.0	1.8	0.4	415.0	—	—	—	46
YSII-SS8-RP2	28.5	71.0	12.9	27.5	4.2	0.4	—	—	—	—	—
Mean	24.8	64.0	10.3	24.3	3.0	0.4	415.0	—	—	—	46
YSII-SS9-LP1	23.0	66.0	12.5	26.5	3.2	0.3	188.0	226.0	247.4	78	86
YSII-SS9-RP1	29.0	72.0	11.0	43.0	1.6	0.4	172.0	211.0	—	74	3
YSII-SS9-LP2	34.0	104.0	11.5	—	—	0.3	180.0	257.0	275.2	41	73
YSII-SS9-RP2	48.0	78.0	17.2	39.0	1.1	0.6	364.0	331.0	—	62	1
YSII-SS9-LP3	30.0	68.5	7.6	—	—	0.4	246.0	—	—	—	—
YSII-SS9-RP3	33.0	84.0	10.3	44.0	4.6	0.4		367.0	—	—	70
YSII-SS9-LP4	—	—	—	—	—	—	—	—	—	—	—
YSII-SS9-RP4	30.5	91.0	6.6	—	—	0.3	—	—	—	—	—
Mean	32.5	80.5	11.0	38.1	2.6	0.4	230.0	278.4	261.3	64	47

Abbreviations: L: Track length; W: Track width; D: Track depth; M'L: Mound length; M'D: Mound depth; L/W: Track length/track width; PL: Pace length; SL: Stride length; TW: Trackway width; PA: Pace angulation; R: Rotation.

Supplementary material. 2. The Photograph and outline drawing of the best-preserved sauropod trackway YSII-SS1. (Photographs and line drawing by L.X.)

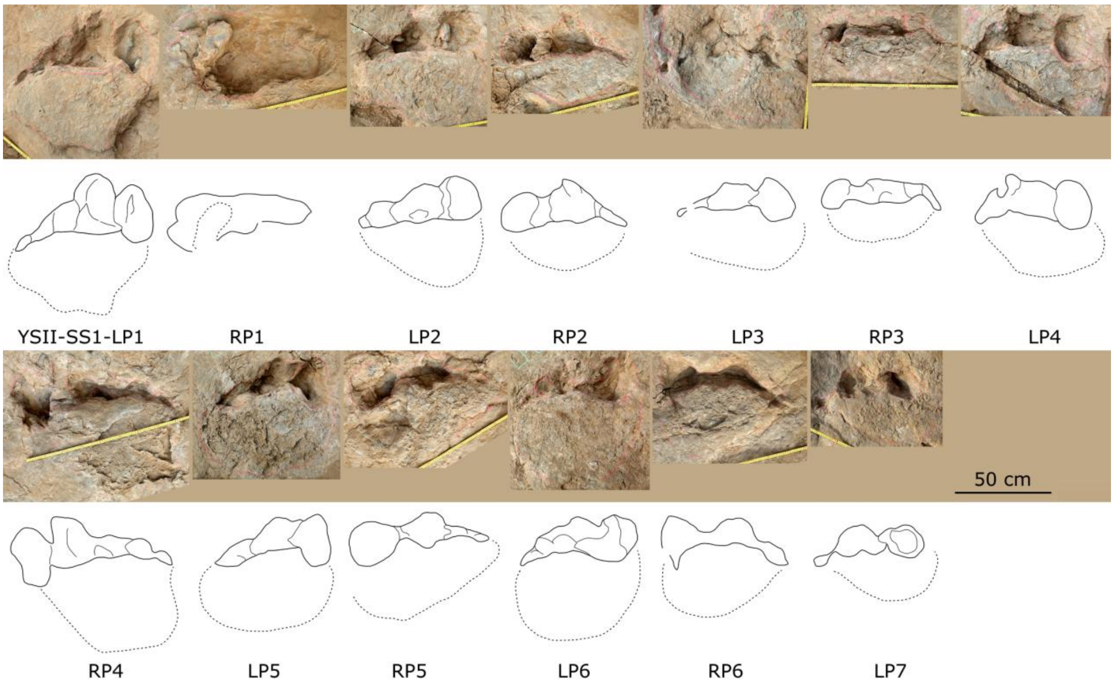

Supplement: Supplementary Information [file srep21138-s2.pdf]
